# Effect of ethanol-treated mid-peripheral epithelium on corneal wound healing in rabbits

**DOI:** 10.1186/1471-2415-13-27

**Published:** 2013-07-03

**Authors:** Hyung Bin Hwang, Tae Hoon Oh, Hyun Seung Kim

**Affiliations:** 1 Department of Ophthalmology, Yeouido St. Mary’s Hospital, College of Medicine, The Catholic University of Korea, Seoul, Korea; 2 Saevit Eye Hospital, Goyang, Gyeonggi, Korea

**Keywords:** Corneal wound healing, Ethanol, VEGF, EGF

## Abstract

**Background:**

To determine the effect of an ethanol-treated mid-peripheral epithelium on wound healing of the corneal epithelium.

**Methods:**

Epithelial removal was performed on 18 rabbit eyes, which were divided into three groups of six eyes each as follows: group 1, an 8.0-mm diameter treated with balanced salt solution (BSS) and an 8.0-mm removal; group 2, an 8.0-mm diameter treated with 20% ethanol for 30 seconds and an 8.0-mm removal; and group 3, a 9.0-mm diameter treated with ethanol and an 8.0-mm removal (barrier zone setting group). The corneal defect area was analyzed post-operatively. The concentrations of vascular endothelial growth factor (VEGF) and epidermal growth factor (EGF) in tears were determined pre-operatively and post-operatively. Healed corneal tissues were examined with light and electron microscopy. Immunohistochemical analysis was also performed to estimate the expression of EGF receptors in healed corneal tissue.

**Results:**

The epithelial healing rate in group 3 was faster than that in the two other groups (p < 0.05). The expression of VEGF and EGF in group 3 was higher than that in the other two groups (p < 0.05). Light microscopy revealed clear healing of the corneal epithelium in all groups except for some cases in group 1. Electron microscopy revealed a relatively intact microstructure of the healed corneal tissues, especially in group 2 and 3 when compared with group 1. Meanwhile, in the immunohistochemistry, group 3 showed significantly higher expression of EGFR when compared with the other groups. Furthermore, EGFR expression had a tendency to be stronger in the mid-peripheral corneal area than in the central corneal area.

**Conclusions:**

The preserved mid-peripheral epithelial layer treated with ethanol (barrier zone) promoted corneal epithelial healing. It appeared to be correlated with elevated tear VEGF and EGF levels in the post-operative period.

## Background

In the field of corneal surgery, 20% dilute ethanol has been used in laser-assisted subepithelial keratectomy (LASEK) to effectively create a corneal epithelial flap. In a LASEK procedure, 20% ethanol treatment for 20–40 seconds separates the epithelium and stroma between the basement membrane or Bowman’s layer level without mechanical trauma on the stromal surface. However, dilute ethanol also induces apoptosis of keratocytes and epithelial cells, which liberates many inflammatory growth factors, such as vascular endothelial growth factor (VEGF) and epidermal growth factor (EGF) [1-4]. VEGF has long been known to be a potent stimulator of the proliferation of blood vessels. VEGF is an endothelial mitogen, an angiogenic factor, and a potent mediator of vascular permeability [5,6]. However, VEGF is also a corneal wound healing mediator. Recently, Lazarovici *et al*. [7] and Nico *et al*. [8] suggested the existence of a paracrine loop between nerve growth factor (NGF) and VEGF. Yu *et al*. [9] reported that VEGF plays a role in mediating corneal nerve repair. Kim *et al*. [10] hypothesized that VEGF has a positive effect on corneal wound healing through a paracrine loop between NGF and VEGF. EGF is a pleiotropic cytokine that can stimulate proliferation, migration and adhesion of corneal epithelial cells during wound healing [11,12]. The EGF receptor has an important role in cell proliferation and stratification, namely maintaining normal corneal thickness after corneal epithelial wounding [13,14].

Meanwhile, there have been various studies on the effect of dilute ethanol on corneal wound healing [15-17]. These studies suggested that ethanol treatment induces the expression of many growth factors and has some positive effect on corneal wound healing. Nevertheless, a study involving the corneal wound healing rate as a function of tear inflammatory cytokine expression has not been reported. To investigate the effect of an ethanol soaked corneal epithelium, we treated rabbit corneas with ethanol and evaluated the effect of the preserved mid-peripheral epithelial layer on wound healing through analysis of the epithelial defect area and tear inflammatory cytokine expression.

## Methods

### Animals

Eighteen eyes of 18 New Zealand white male rabbits weighing 2.0 kg were used in this study. The protocol was approved by the Institutional Animal Care and Use Committee of the Catholic University of Korea (No. 2010-0077-01) and was in accordance with the Association for Research in Vision and Ophthalmology Statement for the Use of Animals in Ophthalmic and Visual Research. All animals were housed in individual cages and maintained under standard conditions in the animal facilities of the Catholic University of Korea.

### Division of groups

**Figure 1:**
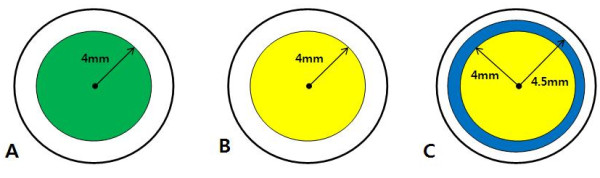
**Illustration of the epithelial removal method in groups 1–3.** Green indicates mechanical epithelial removal. Yellow indicates ethanol-assisted epithelial removal. Blue indicates the barrier zone; that is, an ethanol-treated but not removed area. Epithelial removal size was standardized as an 8.0 mm in diameter in all three groups.

Eighteen rabbits (18 eyes) were randomly divided into three groups of six eyes each according to ethanol treatment and epithelial removal methods as follows: in group 1, the central corneal epithelium (8.0 mm in diameter) was treated with a balanced salt solution (BSS^®^, S.A. Alcon-Couvreur N.V., Puurs, Belgium) and was then removed with a Crescent blade^®^ (Alcon Surgical, Fort Worth, Texas, USA) under the operating microscope after being circumscribed with an 8.0-mm trephine blade; in group 2, the central corneal epithelium (8.0 mm in diameter) was treated with ethanol and then the 8.0-mm portion was removed; and in group 3, the central corneal epithelium (9.0 mm in diameter) was treated with ethanol and then a 8.0-mm central portion was removed. The epithelial removal size was standardized as an 8.0 mm in diameter in all three groups. A balanced salt solution or 20% ethanol was applied for 30 seconds. In group 3, the barrier zone of the cornea (ranging from 8.0–9.0 mm) was treated with ethanol, but was not removed (Figure 1).

### Removal of the corneal epithelium

All procedures were performed with animals under general anesthesia, which was induced by 0.2 mg/kg of Zoletil^®^ intramuscularly (250 mg of Tiletamine and 250 mg of Zolazepam; Vibrac, Carros, France). A wire lid speculum was used to separate the eyelids after applying 0.5% proparacaine hydrochloride (Alcaine^®^; Alcon, Fort Worth, TX, USA). According to the groups, an 8.0-mm Trephine blade^®^ (Katena, Denville, NJ, USA) was placed and fixed firmly on the corneal surface with gentle pressure. A few drops of BSS or ethanol were put into the well and left in place for 30 seconds, then absorbed with a dry cellulose sponge (Merocel^®^; Medtronic Solan, Jacksonville, FL, USA) and irrigated thoroughly with normal saline. The epithelium was peeled using a Crescent blade along the boundary. All procedures of epithelial removal were performed on the right eyes of experimental animals.

### Quantification of the defect area in the corneal epithelium

Through a method of fixed-focus photography, digital photographs were obtained pre-operatively and 1 day, 3 days, 5 days and 7 days after epithelial removal using a camera on a surgical microscope (S21; Carl Zeiss, Jena, Germany). For minute observation of the margin of the corneal wound, a few drops of 2% fluorescein solution without preservatives were applied to the cornea. Changes in the epithelial defect area were calculated with comparison of the area of the corneal wound immediately after epithelial removal and these values were expressed as percent values based on the following formula:

(defect area [pixels] at follow up) / (defect area [pixels] immediately after epithelial removal) × 100 = % ratio of mean area [pixels].

The area of the corneal wound was measured in pixels. Each photograph was analyzed at the same magnification using image analysis software (Image J 1.40 g; Wayne Rasband at the Research Services Branch, National Institutes of Health, Bethesda, MD, USA). Pictures were numbered randomly for data analysis to minimize observer bias. Pathologic lesions, such as inflammation, opacities and new vessel formation were recorded during the course of the study.

### Immunoassay of VEGF and EGF in tear samples

Tears were collected pre-operatively and at 1 day, 3 days and 5 days post-operatively from the lower conjunctival sacs without topical anesthesia using scaled 20-μL microcapillary tubes. An attempt was made to collect the tear fluid samples without light and mechanical stimulation. Tear samples were centrifuged for 5 min (3000 g) at 4°C and the supernatants were collected and transported in an insulated cooler to a -70°C freezer, where the supernatants remained frozen until use in the immunoassay. The VEGF and EGF levels in these samples were analyzed using a Milliplex™ MAP Human Cytokine Kit (Millipore Corporation, St. Charles, MI, USA) and a Luminex 100™ analyzer (Luminex Corp., Austin, TX, USA). The median fluorescence intensity (MFI) of the samples was measured and a standard curve was acquired using Masterplex™ QT (version 4.0; MiraiBio, Inc., Alameda, CA, USA). The absolute concentrations of VEGF and EGF were calculated from standard curves.

### Light and electron microscopy

Harvesting the corneas was performed by sacrificing the animal with an intravenous injection of pentobarbital sodium and extracting the corneal buttons atraumatically. Halves of the corneal buttons were fixed in 4% formaldehyde for 24 hours and embedded in paraffin. The embedded tissues were sectioned to a thickness of 5 μm using a microtome and stained with hematoxylin-eosin. Then, the sections were observed using an Axioimager A1 light microscope (Zeiss, Oberkochen, Germany). The other halves of the corneal buttons were prepared for transmission electron microscopy (TEM) and scanning electron microscopy (SEM). For TEM, the specimens were fixed in 2.5% glutaraldehyde for 24 hours, washed in phosphate buffer (pH 7.4), post-fixed in 1% osmium tetroxide in phosphate buffer (pH 7.4), and dehydrated in increasing concentrations of alcohol. The corneas were then washed with propylene oxide and embedded in epoxy resin-embedding media. Ultrathin sections (60 nm thick) were cut with a glass knife on a LKB ultramicrotome (LKB, Bromma, Sweden). These sections were collected on copper grids, stained with uranyl acetate and lead citrate, and examined with a JEOL-1010 transmission electron microscope (JEOL, Tokyo, Japan). For SEM, fixed corneas were dehydrated in increasing concentrations of acetone. The fixed corneas were critical-point dried, mounted on metal stubs with conductive silver paint, and then sputtered with a 10-nm thick layer of gold in an ion sputter (JFC-1100; JEOL). The corneas were examined with a JSM-5410LV scanning electron microscope (JEOL) at an acceleration voltage of 15 kV.

### Immunohistochemistry for the EGF receptor

Tissue sections (5 μm) were made from paraffin-embedded blocks of corneal buttons. The tissue sections were deparaffinized with xylene, rehydrated in serial ethanol solutions, and then immersed in 0.6% hydrogen peroxide to block endogenous peroxidase. Protein kinase K was used for antigen retrieval. The slides were incubated at 4°C overnight with primary antibody (mouse monoclonal anti-EGFR antibody 31G7, 28–0005, 1:100; Zymed, San Francisco, CA, USA). Goat anti-mouse HRP-conjugated immunoglobulin (Dakocytomation Envision Plus System, Carpinteria, CA, USA) was used as a secondary antibody. The immunoreaction was visualized with 3, 3’-diaminobenzidine (DAB). Counterstaining was performed with Mayer’s hematoxylin. Light microscopy was used for observation. The staining results were scored semiquantitatively as follows: 0, no membranous staining in any of the cells; 1+, weak intensity membranous and cytoplasmic staining of nearly equal intensity; 2+, moderate-to-strong intensity staining predominantly in the membranes; and 3+, strong intensity staining clearly localized to the cell membranes [18].

### Statistical analysis

All data are expressed as the mean ± standard deviation (SD) and were analyzed using SPSS 12.0 for Windows statistical software (SPSS, Inc., Chicago, IL, USA). Pre-operative basal levels of VEGF and EGF were examined with a Kruskal-Wallis test. A repeated measures analysis of variance (RMANOVA) was used for the repeated measures of defect areas and VEGF and EGF quantities. The Mann–Whitney U test was used for intergroup comparisons because *post-hoc* analysis was not established in these non-parametric data. A p value < 0.05 was considered statistically significant.

## Results

### Analysis of defective areas of the corneal epithelium

**Figure 2:**
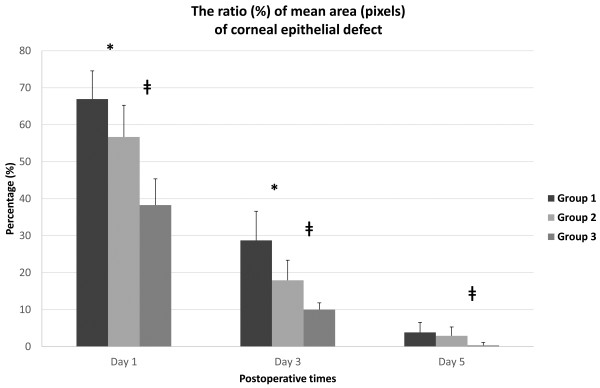
**The ratio (%) of the mean area (pixels) of the epithelial defect measured in each group compared with the original epithelial removal.** The differences between intergroups were statistically significant on post-operative day 1, day 3 and day 5 (p < 0.05, Mann–Whitney U test), but the differences between group 1 and group 2 were not statistically significant on post-operative day 5 (p = 0.394, Mann–Whitney U test). * denotes a statistically significant difference between group 1 and group 2 (p < 0.05). ǂ denotes the difference between group 2 and group 3 (p < 0.05). Group 1: 8.0-mm BSS treatment and 8.0-mm removal, Group 2: 8.0-mm ethanol treatment and 8.0-mm removal, Group 3: 9.0-mm ethanol treatment and 8.0-mm removal.

**Figure 3:**
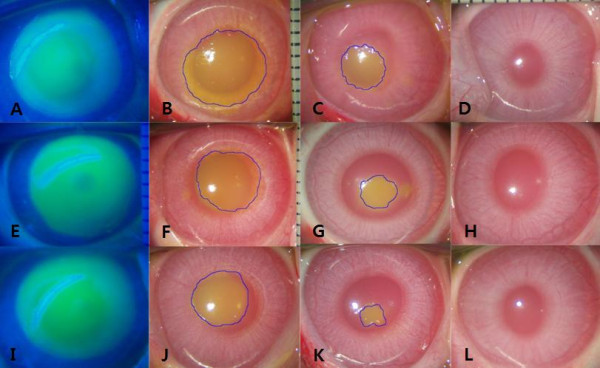
**Representative images of microscopic examination of the unhealed area in the cornea.** Initial defect areas of the corneas immediately after epithelial removal (8.0 mm in size) were observed under cobalt blue lights (**A**, **E**, **I**). One day later, the defect area was markedly decreased in group 3 (**J**), less decreased in group 1 (**B**) and moderately decreased in group 2 (**F**). After 3 days, the defect areas were decreased in all three groups, but in group 1 and group 2 (**C**, **G**), the areas were larger than in group 3 (**K**). On post-operative day 7, the corneas healed to transparency without inflammation or scar formation in all three groups (**D**, **H**, **L**). Group 1: 8.0-mm BSS treatment and 8.0-mm epithelial removal (**A**, **B**, **C**, **D**), Group 2: 8.0-mm ethanol treatment and 8.0-mm epithelial removal (**E**, **F**, **G**, **H**), Group 3: 9.0-mm ethanol treatment and 8.0-mm epithelial removal (**I**, **J**, **K**, **L**).

Five days post-operatively, whole corneas were healed with the exception of one eye each in group 1 and group 2. These two corneas were healed by 7 days post-operatively. There were no pathologic lesions such as inflammation or opacities. The ratio of the mean defective area to the mean area immediately after epithelial removal is demonstrated in Figure 2. The changes in the mean defective area with the course of time were significantly different among the three groups (p < 0.05, RMANOVA). Statistically significant differences were observed between the intergroups at 1 day, 3 days and 5 days post-operatively (p < 0.05, Mann–Whitney U test), except between group 1 and group 2 (p = 0.394, Mann–Whitney U test) at 5 days post-operatively (Figure 2). Thus, the defective areas for group 3 were significantly smaller than those for group 1 and group 2 on post-operative day 1, day 3 and day 5. The defective areas for group 1 were significantly larger than those for group 2 and group 3 on post-operative day 1 and day 3 (Figure 3).

### Analysis of the tear VEGF concentration

**Figure 4:**
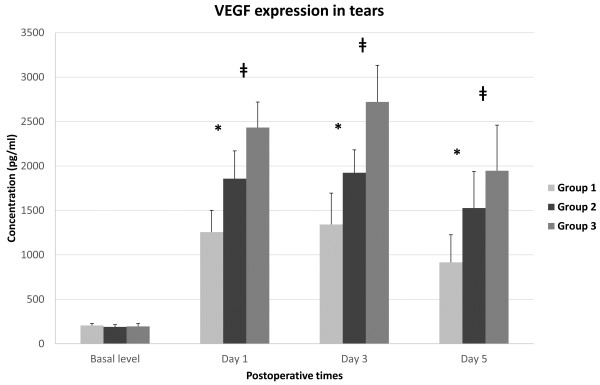
**The concentration (pg/ml) of VEGF in rabbit tears preoperatively 1 day, 3 days and 5 days after epithelial removal.** The differences between groups were statistically significant on post-operative day 1, day 3 and day 5 (p < 0.05, Mann–Whitney U test). * denotes a statistically significant difference between group 1 and group 2 (p < 0.05). ǂ denotes the difference between group 2 and group 3 (p < 0.05). Group 1: 8.0-mm BSS treatment and 8.0-mm epithelial removal, Group 2: 8.0-mm ethanol treatment and 8.0-mm epithelial removal, Group 3: 9.0-mm ethanol treatment and 8.0-mm epithelial removal.

The pre-operative and post-operative expressions of tear VEGF are shown in Figure 4. Pre-operatively, there was no significant difference in the mean tear fluid VEGF levels in eyes randomized by the three groups (p = 0.943, Kruskal-Wallis test). The changes in tear VEGF levels over the course of time were significantly different among the three groups (p < 0.05, RMANOVA). Statistically significant differences were observed between intergroups on post-operative day 1, day 3 and day 5 (p < 0.05, Mann–Whitney U test). Thus, post-operatively, the values for group 3 were significantly higher those for than group 1 and group 2 on post-operative day 1, 3 and 5. Although the tear levels of VEGF decreased after the 3rd post-operative day in the 3 groups, the values for group 3 were still significantly higher than those for the other groups on post-operative day 5 (Figure 4).

### Analysis of the tear EGF concentration

**Figure 5:**
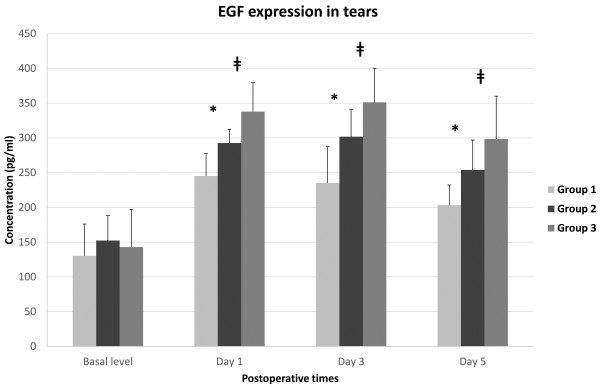
**Concentration (pg/ml) of EGF in rabbit tears preoperatively 1 day, 3 days and 5 days after epithelial removal.** The differences between intergroups were statistically significant on post-operative day 1, day 3 and day 5 (p < 0.05, Mann–Whitney U test). * denotes a statistically significant difference between group 1 and group 2 (p < 0.05). ǂ denotes the difference between group 2 and group 3 (p < 0.05). Group 1: 8.0-mm BSS treatment and 8.0-mm epithelial removal, Group 2: 8.0-mm ethanol treatment and 8.0-mm epithelial removal, Group 3: 9.0-mm ethanol treatment and 8.0-mm epithelial removal.

The pre- and post-operative expressions of tear EGF are shown in Figure 5. Pre-operatively, there were no significant differences in the mean tear fluid EGF levels in the eyes randomized by the three groups (p = 0.591, Kruskal-Wallis test). The changes in tear EGF levels over the course of time were significantly different among the three groups (p < 0.05, RMANOVA). Statistically significant differences existed between the intergroups on post-operative day 1, day 3 and day 5 (p < 0.05, Mann–Whitney U test). Thus, these values for group 3 were significantly higher than those for group 1 and group 2 on post-operative day 1 and day 3. Although the tear levels of EGF decreased after the 3rd post-operative day in the three groups, the values for group 3 were still significantly higher than those for the other two groups on post-operative day 5 (Figure 5).

### Light and electron microscopic findings

**Figure 6:**
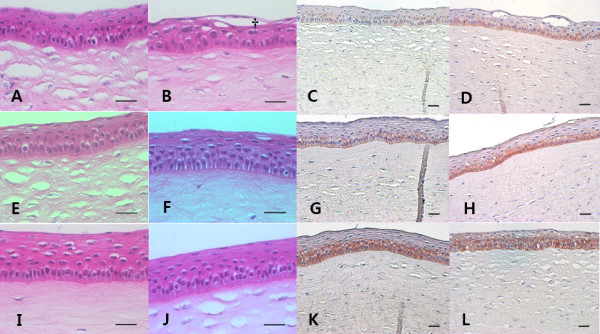
**Representative images of light microscopic examination of healed corneas with hematoxylin-eosin staining (A, B, E, F, I, J) and EGFR immunohistochemistry around the area of central cornea (C, G, K) and mid-peripheral cornea (D, H, L).** The epithelial cell layer was densely packed, but the cells of the superficial layer were not flattened in group 1 (**A**). One case involving the epithelial layer of group 1 (**B**) was less compact than the other groups and showed some empty spaces (crucifix). The epithelial layers of group 2 and group 3 (E, F, I, J) were smooth and densely packed, and the superficial cells were flattened sufficiently. The immunohistochemical score of EGFR was 1+ (weak intensity membranous and cytoplasmic staining of nearly equal intensity) in group 1 (**C**, **D**). The score of EGFR was about 1+ to 2+ (moderate-to-strong intensity staining predominantly in the membranes) in group 2 (**G**, **H**). However, the intensity of EGFR staining in group 3 (**K**, **L**) was stronger than group 1 and group 2. Additionally, the intensity around the area of the mid-peripheral cornea (**D**, **H**, **L**) was stronger than that around the area of the central cornea (**C**, **G**, **K**). In group 3 in particular, the expression of EGFR was spread out nearly along the entire epithelial layer rather than the basal layer (**L**). Group 1: 8.0-mm BSS treatment and 8.0-mm epithelial removal (**A**, **B**, **C**, **D**), Group 2: 8.0-mm ethanol treatment and 8.0-mm epithelial removal (**E**, **F**, **G**, **H**), Group 3: 9.0-mm ethanol treatment and 8.0-mm epithelial removal (**I**, **J**, **K**, **L**); Scale bar – 50 μm.

Histopathologic assessment of corneal tissues by light microscopy revealed a clearly healed and stratified epithelium without any complications, such as inflammation and opacities, in all three groups (Figure 6). However, in group 1, the epithelial cells of the superficial layer were not flattened sufficiently and one case of a loose epithelial arrangement was found.

**Figure 7:**
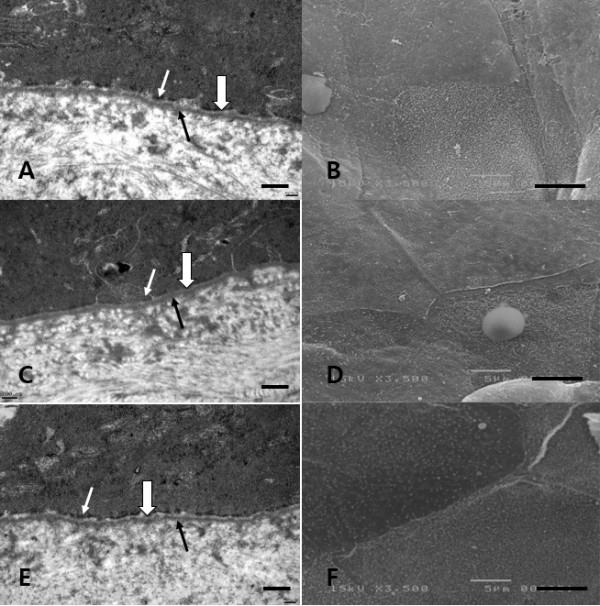
**Representative images of transmission electron micrographs (TEM, ×40,000) of the basement membrane zone and scanning electron micrographs (SEM, ×3,500) of the superficial epithelium and microvilli of healed rabbit corneas on postoperative day 7.** TEM: The lamina lucida (empty arrow) and lamina densa (black arrow) of the basement membrane zones were relatively well-preserved without disruption or undulation of the layers in all three groups, and the basement membrane zones were lined by electron-dense hemidesmosomes (white arrow; **A**, **C**, **E**). SEM: Epithelial cellular structure and microvilli were restored relatively normally without a definite injury in group 2 (**D**) and group 3 (**F**). However, a somewhat injured epithelial cellular structure was observed in group 1 (**B**). Group 1: 8.0-mm BSS treatment and 8.0-mm epithelial removal (**A**, **B**), Group 2: 8.0-mm ethanol treatment and 8.0-mm epithelial removal (**C**, **D**,) Group 3: 9.0-mm ethanol treatment and 8.0-mm epithelial removal (**E**, **F**); Scale bar – 1 μm (**A**, **C**, **E**) and 5 μm (**B**, **D**, **F**).

SEM revealed a relatively normal epithelial cellular regeneration and an ongoing recovery of microvilli in the healed corneal tissues of group 2 and group 3. However, a somewhat injured epithelial structure was observed in the healed corneal tissues of group 1. TEM revealed a sharp basement membrane structure composed of lamina lucida and lamina densa with some hemidesmosomes in all three groups (Figure 7).

### Immunohistochemistry for the EGF receptor

Seven days post-operatively, the expression of EGFR was fulfilled predominantly in the basal epithelial cell layer. The immunohistochemical score of EGFR was approximately 1+ in group 1, and the score was about 1+ to 2+ in group 2. Additionally, the intensity of EGFR staining was stronger in group 3 than that in group 1 and group 2, and the score was about 2+ to 3+. In general, the intensity around the mid-peripheral corneal area showed a strong tendency compared with intensity around the central corneal area in all groups. In group 3 in particular, the expression of EGFR was spread out nearly along the entire epithelial layer rather than the basal layer (Figure 6).

## Discussion

Generally, within minutes after a corneal epithelial injury, cells from the limbus at the edge of the abrasion begin to migrate centripetally to cover the defect rapidly at a rate of 60–80 μm per hour. A variety of peptide growth factors, such as EGF, VEGF, TGF-ß, fibronectin and fibrinogen, appear to play important roles in the regulation of normal corneal wound healing [19-21]. In the current study, the ethanol-treated barrier zone group (group 3) induced increased tear VEGF and EGF expression compared with the mechanical stripping group (group 1) and the ethanol-assisted stripping group (group 2). It appeared that VEGF and EGF released from the ethanol-treated barrier zone group (group 3) especially promoted epithelial wound healing. In addition, a barrier zone did not exert tissue injuries. We could confirm this point by observing the light microscopic findings. Furthermore, electron microscopic findings revealed normal basement membrane structures, normal microvilli and epithelial regeneration when compared with group 1 and group 2. In addition, group 3 showed significantly stronger expression of EGFR in the immunohistochemistry results when compared with the other groups at the end of the wound healing process. Therefore, we reasoned that an ethanol-treated epithelial layer in the barrier zone promoted epithelial wound healing via increased expression of VEGF and EGF. In fact, VEGF exerts a positive effect on epithelial wound healing through the effect of NGF [7-10]. Meanwhile, the expression of EGFR was revealed to be higher in the area of the mid-peripheral cornea than in the area of the central cornea. Therefore, we could postulate that the signal of wound healing manifested more strongly in the peripheral area than in the central area of the cornea.

Our results indicated that the mechanical stripping method (group 1) induced decreased expression of tear VEGF and EGF expression compared with the ethanol-assisted stripping method (group 2) and barrier zone group (group 3). Therefore, group 1 had a slower epithelial healing rate and unstable histologic findings in view of light and electron microscopic findings compared with group 2 and group 3. In other words, some cases in group 1 could not be clearly healed of superficial epithelium compared with group 2 and group 3. However, we think that there would be another reason for slower wound healing in group 1. A dilute ethanol treatment is known to separate the epithelium and stroma between the basement membrane or Bowman’s layer. Because the mechanical stripping method did not result in a smooth stromal bed, this method could not lead to fast epithelial healing. Thus, ethanol treatment during LASEK surgery plays a role of not only stimulating corneal wound healing, but also creating a smooth stromal bed and minimizing mechanical corneal injuries caused by epithelial stripping and laser ablation.

Our study suggested that the ethanol-treated barrier zone increases expression of inflammatory growth factors, such as VEGF and EGF, thus promoting corneal epithelial healing. Similarly, there are various studies regarding the effect of dilute ethanol on corneal wound healing. Miyamoto *et al*. [15] reported that dilute ethanol exposure activated nuclear factor kappa-light-chain-enhancer of activated B cells (NF-kB) and up-regulated cyclooxygenase (COX)-2 expression in a rat corneal model. Furthermore, Miyamoto *et al*. [15] demonstrated that ethanol treatment increased inflammatory reactions because COX-2 and NF-kB activated MMPs and VEGF, respectively. Kim *et al*. [16] reported the proliferation of epithelial cells when 20% dilute ethanol-treated cells were added to the culture medium of porcine corneal epithelial cells. Kim *et al*. [16] concluded that ethanol-treated epithelial cells released certain growth factors that might be involved in cell proliferation, and emphasized the need to identify the factors. Therefore, we thought that VEGF and EGF were a part of these factors involved in cell proliferation. Okada *et al*. [17] investigated the pattern of expression of c-Fos, the major component of activator protein (AP)-1, in rat corneal epithelium exposed to dilute ethanol. c-Fos protein was found in the corneal epithelium around the area of ethanol exposure. No immunoreactivity for this protein was detected in the area of ethanol exposure. Okada *et al*. [17] suggested that the strong epithelial cell c-Fos protein expression outside the ethanol-exposed area played a role in epithelial repair in response to epithelial cell death in the central area because the area of dead cells was replaced by migratory surrounding cells. The three above-mentioned studies confirmed that ethanol treatment has a positive effect on corneal wound healing. Moreover, the findings in the current study are in agreement with these three reports.

Our study has some limitations that lessen the applicability of the results to clinical situations. In other words, more biological experiments will be needed to discover the detailed mechanisms of the stimulatory effect of the mid-peripheral barrier zone on epithelial wound healing. The study on cell migration and proliferation will be especially valuable to discover the mechanism. TGF-β is well known to enhance migration of the corneal epithelium during wound healing; that is, during healing of corneal epithelial defects, endogenous TGF-β activates p38 mitogen-activated protein kinase (MAPK) for cell migration and suppression of cell proliferation, resulting in rapid initial resurfacing of the epithelium in the early healing phase [22,23]. Meanwhile, it has also been demonstrated that inhibition of p38MAPK activation enhances EGF and keratocyte growth factor (KGF) stimulation of Erk-1/2 activity. This MAPK-Erk pathway is known to accelerate cell proliferation at the late healing phase [22,24,25]. Therefore, biological strategies, such as immunoassay and immunostaining for TGF-β-p38MAPK signal and the MAPK-Erk pathway, are helpful to prove the stimulatory effect of the barrier zone group on corneal wound healing. In addition, epithelial VEGF and EGF should be analyzed in future studies. We analyzed only tear VEGF and EGF to observe the progress of cytokine expression with the course of time within the same individual animal without sacrificing it. However, tear VEGF and EGF levels are different with cytokines expressed from corneal epithelial cells and keratocytes in certain aspects. Finally, we only analyzed VEGF and EGF, but neglected other important growth factors and cytokines that influence the healing process after excimer laser ablation. Further factors, such as fibronectin, fibrinogen, should be analyzed in future studies.

## Conclusions

In conclusion, we suggest that ethanol-treated mid-peripheral corneas promote wound healing of the central corneal epithelium, and increase VEGF and EGF expression in tears. Therefore, ethanol treatment could be a positive factor in corneal wound healing. The stronger expression of EGFR around the mid-peripheral corneal area compared with the central corneal area supports this hypothesis. Furthermore, the ethanol-treated barrier zone does not exert a harmful influence on the microstructure of corneas, such as basement membrane disruption and microvilli damage. In our submission, dilute ethanol treatment on the cornea seems to trigger a positive effect on corneal wound healing.

## Competing interests

The authors declare that they have no conflict of interests.

## Authors’ contributions

Literature screening and selection was performed by HBH, THO and HSK. The animal experiment was implemented by HBH and THO. HBH, THO and HSK participated in the design of the study. HBH performed the statistical analyses. Preparation of the first draft of the manuscript was done by HBH, and review and approval of the manuscript was performed by THO and HSK. All authors read and approved the final manuscript.

## Pre-publication history

The pre-publication history for this paper can be accessed here:

http://www.biomedcentral.com/1471-2415/13/27/prepub
